# Mitral annular disjunction in surgical mitral valve prolapse: prevalence, characteristics and outcomes

**DOI:** 10.1186/s44156-023-00032-x

**Published:** 2023-11-08

**Authors:** Rhys Gray, Praveen Indraratna, Gregory Cranney, Hebe Lam, Jennifer Yu, Gita Mathur

**Affiliations:** 1 Department of Cardiology, Prince of Wales Hospital, Sydney, NSW Australia; 2 University of New South Wales, Sydney, Australia

**Keywords:** Mitral valve prolapse, Mitral annular disjunction, Mitral valve repair, Mitral, Valve replacement

## Abstract

**Background:**

There is a paucity of literature regarding outcomes of patients with mitral valve prolapse (MVP) and mitral annular disjunction (MAD) after mitral surgery, with many unanswered questions including the post-surgical persistence of MAD, effect of MAD on mitral valve reparability, and incidence of arrhythmia after mitral valve surgery. We aimed to examine the prevalence, imaging characteristics and clinical associations of mitral annular disjunction among patients undergoing mitral valve surgery for mitral valve prolapse, as well as outcomes after surgery including the persistence of MAD, arrhythmic events and excess mortality.

**Results:**

A retrospective analysis of 111 consecutive patients who underwent mitral valve surgery for MVP was performed. A total of 32 patients (28.8%) had MAD. Patients with MAD were younger (64 vs 67 yrs, p = 0.04), with lower rates of hypertension (21.9% vs 50.6%, p = 0.01) and hyperlipidaemia (25% vs 50.6%; p = 0.01) and were more likely to be female (43.8% vs 21.4%, p = 0.04) with myxomatous leaflets > 5mm (90.6% vs 15.2%, p =  < 0.01) and bileaflet prolapse (31.3% vs 10.1%, p = 0.02). Mitral valve repair was performed in 29/32 patients (90.6%) in the MAD positive group, and no patients had the persistence of MAD post-surgery. Post-operative ventricular arrhythmia was higher in the MAD positive group (28.13% vs 11.69%, p = 0.04) with no difference in mortality, 30-day hospital re-admission, or post-operative mitral regurgitation between patients with and without MAD over 3.91 years of follow up.

**Conclusion:**

In this study of consecutive patients with MVP undergoing surgery, MAD was a common finding (almost 1 in 3). MAD does not compromise mitral valve surgical reparability, and both repair and replacement are effective at correcting disjunction. Our data suggest that concurrent MAD in MVP patients undergoing surgery has no significant effect on post surgical outcomes. Further research as to whether this patient cohort requires post-surgical arrhythmia monitoring is warranted.

## Introduction

Mitral valve prolapse (MVP) affects 3% of the population and is the leading indication for mitral valve repair [1]. Population and cohort studies have demonstrated that outcomes in MVP are related mainly to patient age, left ventricular ejection fraction, and mitral regurgitation severity [2, 3], with minimal consequence if mitral regurgitation is not significant. Although the association between MVP and sudden cardiac death was first reported decades ago, the risk was initially believed to be exceedingly small [4]. More recent observational studies have found that MVP is associated with malignant ventricular arrhythmias and sudden cardiac death in a subset of young and middle-aged patients [5–8], giving concern there is an arrhythmic MVP variant. Recent studies have yielded new insights into the pathophysiology and risk factors for the development of ventricular arrhythmia in patients with MVP with the goal of improving patient risk stratification [9, 10].

Mitral annular disjunction (MAD) is a structural abnormality with a distinct separation of the mitral valve annulus / left atrium wall and myocardial continuum [11]. It can occur in a 360 degree arc around the mitral valve annulus, but is most often visualized in the region of the infero-lateral myocardium directly under the posterior mitral valve leaflet, typically in the region of the P1 and P2 mitral valve scallops [1]. The diagnosis of MAD can reliably be made on non-invasive imaging modalities including transthoracic echocardiography, transoesophageal echocardiography, and cardiac magnetic resonance imaging [11, 13, 14]. On transthoracic echocardiography, this is most commonly seen in the parasternal long‐axis view and to a lesser extent from the apical four chamber view, three chamber view and two‐chamber view in the systolic phase.

Previous studies have suggested that MAD, in addition to bileaflet MVP and papillary muscle fibrosis, is associated with an independent increased risk of ventricular arrhythmia [7, 8, 15]. It has been proposed that in patients with MAD, the abnormal mitral annular dynamics due to a non-communicating posterior leaflet results in myocardial fibrosis which may be an integral part of the pathophysiology leading to increased arrhythmic risk [16, 17].

The literature regarding the prevalence of MAD in patients with MVP undergoing mitral valve surgery is limited and varies from 16.2 to 31% [18–21]. The phenotypic MVP characteristics associated with MAD also remain uncertain in this patient cohort. There is a paucity of literature regarding outcomes of patients with MVP and MAD after mitral surgery, with many unanswered questions remaining including the post-surgical persistence of MAD, effect of MAD on mitral valve reparability, and incidence of arrhythmia after mitral valve surgery. These are important considerations as the benefit of mitral valve surgery in terms of reducing arrhythmic risk in patients with MVP and MAD is yet to be determined. Thus, we aimed to examine the prevalence, imaging characteristics and clinical associations of MAD among patients undergoing mitral valve surgery for MVP, as well as the persistence of MAD and outcomes after surgery including arrhythmic events and mortality.

## Methods

### Patient selection (inclusion and exclusion criteria)

All consecutive patients > 18yrs of age who underwent mitral valve surgery for MVP between January 2016 and April 2020 at an Australian quaternary state-wide referral centre for cardiothoracic mitral valve surgery were included in the study. Exclusion criteria included previous mitral valve surgery, MVP due to infective endocarditis, moderate or greater aortic stenosis, aortic regurgitation or mitral stenosis, congenital heart disease and poor image quality precluding accurate assessment of the mitral valve. The study was approved by the South Eastern Sydney Local Health District (SESLHD) Research, Ethics and Governance Office and the Human Research Ethics Committee. The study protocol conforms to the ethical guidelines of the 1975 Declaration of Helsinki.

### Demographic and clinical data

Single site demographic and clinical information was collected from the Australia and New Zealand Society of Cardiac and Thoracic Surgeons (ANZSCTS) National Cardiac Surgery Database for mitral valve surgery, which is a prospective Australia-wide registry which captures patient characteristics and 30-day follow up of adult cardiac surgery. 4-year clinical outcomes were obtained by review of medical records and outpatient cardiology letters.

### Echocardiographic evaluation

**Fig. 1:**
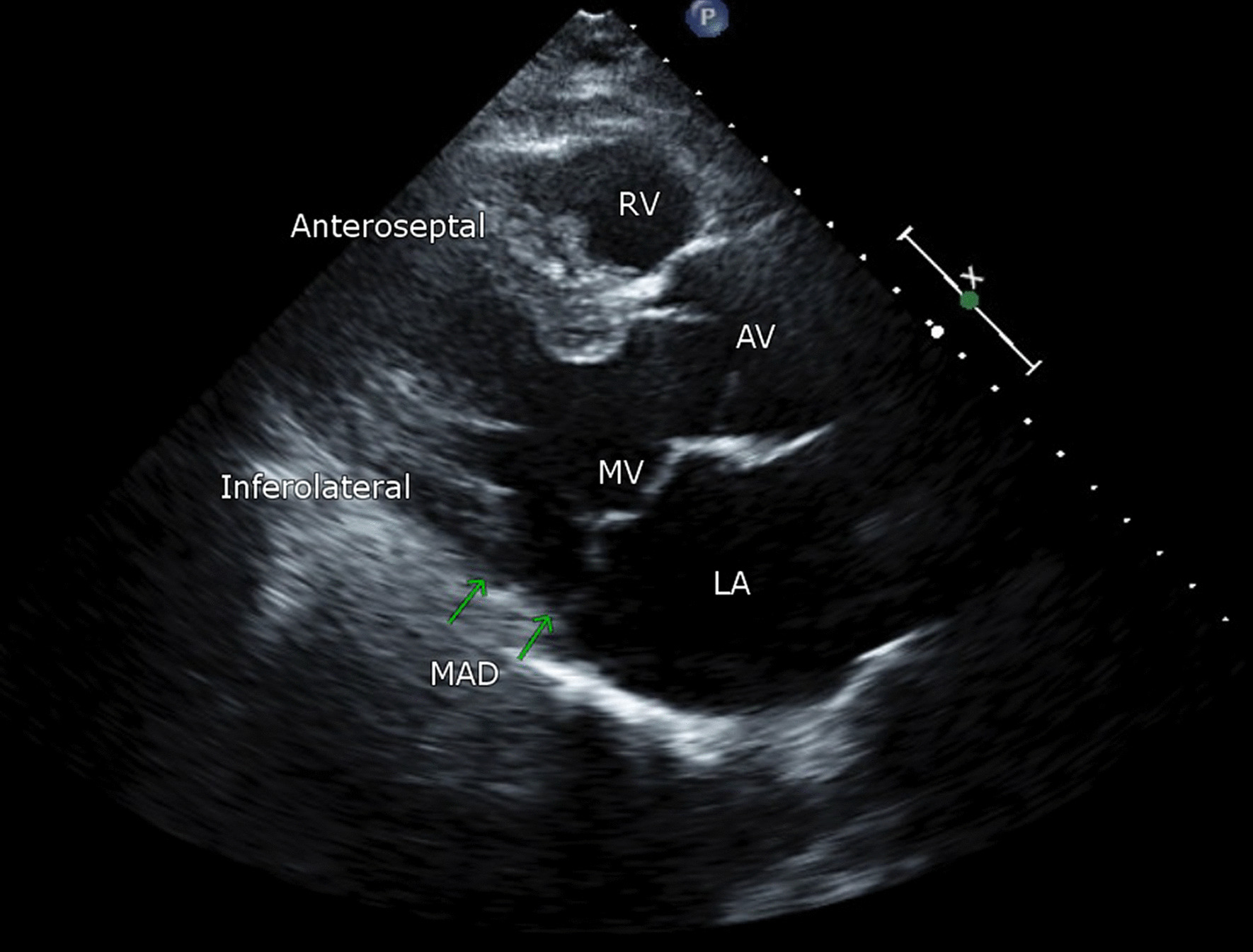
Parasternal long axis view at end systole demonstrating mitral annular disjunction. *RV* right ventricle, *AV*  aortic valve, *MV* mitral valve, *LA*  left atrium, *MAD* mitral annular disjunction

Complete standard 2D and doppler transthoracic echocardiogram protocol was performed according to laboratory practice. All postoperative transthoracic echocardiograms were undertaken in the same laboratory as the pre surgical study. Postoperative studies were performed within 7 days of the index surgery. Left atrial (LA) area and volume were assessed using the modified Simpson’s method in the apical four chamber and two chamber views at end systole. The maximum LA volume indexed to body surface area (LA volume index) was determined. Left ventricular (LV) end-systolic and end-diastolic volumes and ejection fraction (EF) were computed from four-chamber and two-chamber views using the biplane Simpson’s method. MVP was defined as a systolic displacement ≥ 2mm of one or both mitral valve leaflets above the mitral valve annular plane as observed in the parasternal long-axis view. Mitral regurgitation (MR) grade was established integrating qualitative and quantitative criteria according to current ASE guildlines [22]. Mitral valve leaflet length and thickness were measured during diastole in the parasternal long-axis view and was described as myxomatous if leaflet thickness was > 5 mm. The anteroposterior mitral annular diameter was measured in the parasternal long axis view at end-diastole and end-systole. MAD distance was measured in the parasternal long-axis view at end-systole as the distance between the posterior scallop insertion into the LA wall and the systolic bulge of the ventricular myocardium [21] (Fig. 1). A disjunction distance of > 5mm was defined as mitral annular disjunction [13]. There is no universally accepted cut off for MAD, with variability in reporting between studies and modalities. Early histological reports and papers first describing MAD used a cut off of 5mm [10]. We have adopted this as a cut-off to reduce the overreporting of normal/physiological MAD. Pickelhaube sign was defined as a high-velocity spike > 16cm/sec in the tissue doppler velocity profile of the lateral mitral valve annulus.

### Arrhythmia characterisation

The baseline 12-lead electrocardiogram (ECG) at time of surgery was reviewed for all patients. Arrhythmic outcomes (ventricular tachycardia, ventricular fibrillation, frequent ventricular ectopic beats) were recorded by reviewing medical records including daily reviews of inpatient telemetry, outpatient Holter monitor reports and implantable device interrogations where applicable. Ventricular tachycardia (VT) was defined as > 3 beats at a rate of > 120 bpm [23]. Frequent ventricular ectopy was defined as > 7 premature ventricular ectopic beats per min [24]. The composite endpoint of postoperative ventricular arrhythmia included VT on inpatient telemetry, VT or frequent ventricular ectopy on outpatient Holter, VT on device interrogation and in hospital cardiac arrest. The composite endpoint of clinically relevant ventricular arrhythmia included VT on telemetry requiring initiation of antiarrhythmic therapy, sustained VT (> 30 s) on telemetry, non-sustained VT > 10 s on device interrogation and in-hospital cardiac arrest.

### Statistical analysis

Continuous variables are presented as mean ± standard deviation or median (interquartile range) where appropriate. Categorical variable are expressed in absolute numbers and percentages. Patients were split into 2 groups based on the presence or absence of MAD. Differences between MAD positive and MAD negative groups were assessed using two sample t test and wilcoxon rank-sum test for continuous data and pearson’s chi-squared test and fisher’s exact test for categorical data. All statistical analysis were performed using Stata Statistical Software: Release 15 (College Station, TX: StataCorp LLC). For all statistical analyses, a p-value < 0.05 was considered statistically significant.

## Results

### Patient demographics

**Fig. 2:**
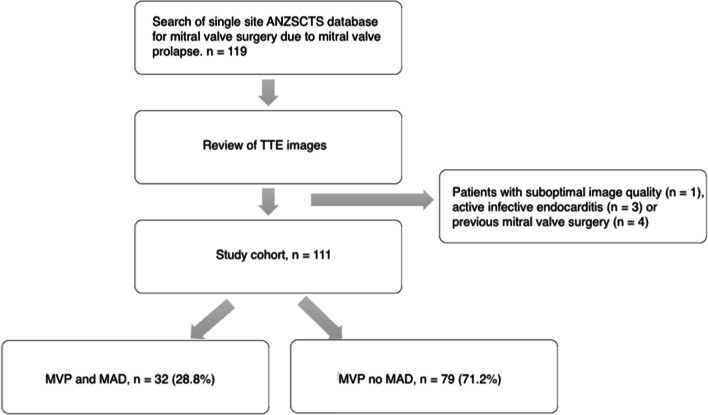
Study flow chart. *POWH *Prince of Wales Hospital; *ANZSCTS *Australian & New Zealand Society of Cardiac & Thoracic Surgeons; *TTE* transthoracic echocardiography; *MVP* mitral valve prolapse; *MAD* mitral annular disjunction

A total of 119 patients were considered for inclusion. Eight patients were excluded, four due to previous mitral valve surgery, three due to active infective endocarditis and one due to significant mitral annular calcification precluding accurate assessment of the mitral valve (Fig. 2). A total of 111 consecutive eligible patients who underwent mitral valve surgery for MVP were included. A total of 32 patients (28.8%) had MAD. Mean age was 66.3 years and 29.7% were female. Baseline demographics and clinical characteristics are presented in Table 1. Patients with MAD were significantly younger (64.0 vs 67.6yrs, p = 0.042), more likely to be female (43.8% vs 21.4%, p = 0.04), and had lower rates of hypertension (21.9% vs 50.6%, p = 0.01) and hyperlipidaemia (25% vs 50.6%; p = 0.01). Only 4/32 (12.5%) patients in the MAD positive cohort had a history of coronary artery disease requiring revascularisation vs 20/79 (25.3%) patients in the MAD negative group, however this did not reach statistical significance (p = 0.14). Only two patients had a history of VT requiring ablation or AICD insertion and both were in the MAD negative cohort. Mitral valve repair was performed in 29/32 patients (90.6%) in the MAD positive group and 60/79 (75.9%) in the MAD negative group, with no significant difference between groups (p = 0.11). Patients with MAD had significantly higher rates of inferior T-wave inversion (28.1% vs 2.5%, p < 0.01) and ventricular ectopic beats (9.4% vs 1.1%, p = 0.02) on baseline 12-lead ECG.

**Table 1:** Patient demographics

	Total (n = 111)	MAD (n = 32)	No MAD (n = 79)	p-value
Age (years)	66.3 (59.5–74.5)	64.0 (52.5–71.8)	67.6 (60.3–75.2)	0.042
Sex (female)	33 (29.7%)	14 (43.8%)	19 (24.1%)	0.040
Current smoker	3(2.7%)	2 6.3%)	1 (1.3%)	0.200
Ex-smoker	46 (41.4%)	11 (34.4%)	35 (44.3%)	0.340
Diabetes	7 (6.3%)	1 (3.1%)	6 (7.6%)	0.670
Hyperlipidaemia	48 (43.2%)	8 (25.0%)	40 (50.6%)	0.014
Dialysis	1 (0.9%)	0 (0.0%)	1 (1.3%)	1.000
Hypertension	47 (42.3%)	7 (21.9%)	40 (50.6%)	0.005
Stroke	7 (6.3%)	0 (0.0%)	7 (8.9%)	0.190
PVD	3 (2.7%)	0 (0.0%)	3 (3.8%)	0.560
Previous MI	3 (2.7%)	0 (0.0%)	3 (3.8%)	0.560
History of CCF	39 (35.1%)	10 (31.3%)	29 (36.7%)	0.590
NYHA class				0.68
1	32 (28%)	11 (34.4%)	21 (26.6%)	
2	51 (45.9%)	15 (46.9%)	36 (45.6%)	
3	21 (18.9%)	4 (12.5%)	17 (21.5%)	
4	7 (6.3%)	2 (6.3%)	5 (6.3%)	
Pre-op AF/Flutter	25 (22.5)	7 (21.9)	18 (22.8)	1.00
Previous CABG	3 (2.7%)	0 (0.0%)	3 (3.8%)	0.56
Previous PCI	6 (5.4%)	1 (3.1%)	5 (6.3%)	0.67
CAD requiring revascularisation	24 (21.6%)	4 (12.5%)	20 (25.3%)	0.14
Concurrent CABG	18 (16.2%)	3 (9.4%)	15 (19%)	0.27
Mitral valve Surgery performed				0.11
Repair	89 (80.2%)	29 (90.6%)	60 (75.9%)	
Replacement	22 (19.8%)	3 (9.4%)	19 (24.1%)	
ECG—inferior TWI	11 (9.3%)	9 (28.1%)	2 (2.5%)	< 0.001
ECG—VEBs	5 (4.5%)	4 (9.4%)	1 (1.1%)	0.007

*PVD* Peripheral vascular disease, *MI* Myocardial infarction, *CCF* Congestive cardiac failure, *NYHA* New York heart association, *AF* Atrial fibrillation, *CABG* Coronary artery bypass grafting, *PCI* Percutaneous coronary intervention, *CAD* Coronary artery disease, *ECG* Electrocardiogram, *TWI* T wave inversion, *VEBs* Ventricular ectopic beats

### Echocardiographic characteristics

Table 2 demonstrates the echocardiographic characteristics of the cohort. A total of 32 patients (28.8%) had MAD with a median disjunction length of 8.5mm. The maximal disjunction length reported was 28mm. When comparing patients with and without MAD. Patients with MAD were more likely to have myxomatous leaflets > 5mm (90.6% vs 15.2%, p =  < 0.01) and bileaflet prolapse (31.3% vs 10.1%, p = 0.02). MAD was associated with longer anterior (31.2mm vs 28.9mm, p = 0.01) and posterior (17.3mm vs 15.8mm, p = 0.01) mitral valve leaflets and larger mitral annular end systolic (35.7mm vs 33.3mm, p = 0.007) and end diastolic (36.8mm vs 32.8mm p =  < 0.01) dimensions. MAD was also associated with a positive pickelhaube sign (34.4% vs 0.0%, p =  < 0.01). MAD patients were more likely to have severe mitral regurgitation compared to MAD negative patients, although this was not statistically significant (59.4% vs 50.6%, p = 0.4). There was no difference between LA volume index (51 ml/m^2^ vs 52ml/m^2^_,_ p = 0.81) and LV end diastolic diameter (58.5mm vs 56.8mm, p = 0.19) between groups. Left ventricular ejection fraction (LVEF) was also similar between the 2 groups, with 90.6% of the MAD positive group and 86.1% of the MAD negative group having a LVEF > 50%. Only one patient had moderate mitral regurgitation post-surgery. The patient with moderate post-surgical mitral regurgitation had MAD with a disjunction length of 7mm, myxomatous bileaflet prolapse and underwent mitral valve repair. No patients had the persistence of MAD post-surgery.

**Table 2:** Echo characteristics

	Total (n = 111)	MAD (n = 32)	No MAD (n = 79)	p-value
BSA	1.97 (.20)	2.02 (.22)	1.95 (.19)	0.10
LA area	28 (24–33)	27 (26–32)	28 (24–33)	0.85
LA volume indexed	52 (44–69)	52 (44.5–64)	51 (44–70)	0.81
LVEDD	57.27 (6.32)	58.50 (5.91)	56.77 (6.45)	0.19
LVESD	32.49 (7.44)	32.16 (8.11)	32.62 (7.19)	0.77
FS	42 (36–48)	42 (38.5–46)	42 (35–49)	0.74
LVED volume	168.87 (35.10)	175.25 (33.57)	166.29 (35.58)	0.22
LVES volume	60 (45–77)	65(51.5–76.5)	59 (45–77)	0.35
EF				
> 50%	97 (87.4%)	29 (90.6%)	68 (86.1%)	1.00
40–50%	8 (7.2%)	2 (6.3%)	6 (7.6%)	
30–40%	5 (4.5%)	1 (3.1%)	4 (5.1%)	
< 30%	1 (0.9%)	0 (0.0%)	1 (1.3%)	
PA pressure	35 (28–46)	34 (30–44.5)	35 (30–46)	0.31
MR severity				0.40
Moderate/severe	52 (46.8%)	13 (40.6%)	39 (49.4%)	
Severe	59 (53.2%)	19 (59.4%)	40 (50.6%)	
Prolapsing leaflet				0.02
Anterior	19 (17.1%)	5 (15.6%)	14 (17.7%)	
Posterior	74 (66.7%)	17 (53.1%)	57 (72.2%)	
Bileaflet	18 (16.2%)	10 (31.3%)	8 (10.1%)	
Anterior leaflet length	29.60	31.22	28.94	< 0.01
Posterior leaflet length	16.23	17.25	15.81	< 0.01
Myxomatous leaflets > 5mm	41 (36.9%)	29 (90.6%)	12 (15.2%)	< 0.01
Mitral annular dimension (antero-posterior) end diastole	33.93 (4.52)	36.78 (4.94)	32.77 (3.79)	< 0.01
Mitral annular dimension (antero-posterior) end systole	33.98 (4.23)	35.66 (4.72)	33.30 (3.84)	< 0.01
Lateral annulus pickelhaube sign	11/87 (9.9%)	11/25 (34.4%)	0/62 (0.00%)	< 0.01
MR severity post op				0.54
Nil	24 (21.6%)	7 (21.9%)	17 (21.5%)	
Trivial	77 (69.4%)	22 (68.8%)	55 (69.6%)	
Mild	9 (8.1%)	2 (6.3%)	7 (8.9%)	
Moderate or greater	1 (0.9%)	1 (3.1%)	0 (0.00%)	
Length of MAD		8.5 (7–11)		
Presence of MAD post op		0 (0.0%)		

*BSA* Body surface area, *LA* Left atrium, *LVED* Left ventricular end diastolic dimension, *LVESD*  Left ventricular end systolic dimension, *EF* ejection fraction, *PA* Pulmonary artery, *MR* Mitral regurgitation, *MAD* Mitral annular disjunction

## Outcomes

Total follow up was 3.91 ± 1.29 years in which there were 5 deaths (4.5%), with 2 deaths (1.8%) occurring in the first 30 days post-surgery. All deaths occurred in the MAD negative group. A total of 11 (9.9%) patients were readmitted in the first 30 days post-surgery and 42 patients (37.8%) developed postoperative atrial fibrillation. The incidence of postoperative ventricular arrhythmia was significantly higher in the MAD positive group (28.13% vs 11.69%, p = 0.04). The incidence of clinically relevant ventricular arrhythmia was also higher in the MAD group, however this did not reach statistical significance (9.4% VS 3.8%, p = 0.35). There was no significant difference in mortality, hospital readmission, postoperative atrial fibrillation or postoperative mitral regurgitation between the MAD positive and MAD negative groups. All outcome data is demonstrated in Table 3.

**Table 3:** Outcomes

	Total n = 111	MAD n = 32	No MAD n = 79	p-value
In hospital mortality	1 (0.9%)	0 (0.0%)	1 (1.3%)	1.0
30 day mortality	2 (1.8%)	0 (0.0%)	2 (2.5%)	1.0
4 year mortality	5 (4.5%)	0 (0.0%)	5 (6.3%)	0.32
30 day hospital readmission	11 (9.9%)	4 (12.5%)	7 (8.9%)	0.73
Post op AF	42 (37.8%)	11 (34.4%)	31 (39.2%)	0.14
In hospital NSVT	14 (12.6%)	6 (18.8%)	8 (10.1%)	0.22
In hospital sustained VT	4 (3.6%)	3 (9.4%)	1 (1.3%)	0.071
In hospital cardiac arrest	1 (0.9%)	0 (0.0%)	1 (1.3%)	1.00
NSVT on outpatient holter	7/42	4/12	3/30	0.088
Frequent VEB on outpatient holter	2/42	1/12	1/30	0.50
NSVT on device interrogation	1/15	0/2	1/13	1.00
Post-operative ventricular arrhythmia*	18 (17.1%)	9 (28.1%)	9 (11.69%)	0.035
Clinically relevant ventricular arrhythmia**	6 (5.4%)	3 (9.4%)	3 (3.8%)	0.35
Follow up (years)	3.91 ± 1.29	3.90 ± 1.37	3.92 ± 1.27	

*AF* Atrial Fibrillation, *NSVT* Non sustained ventricular tachycardia, *VT* Ventricular tachycardia, *VEB* Ventricular ectopic beat

^*^The composite endpoint of post-operative ventricular arrhythmia included VT on inpatient telemetry, VT or frequent ventricular ectopy on outpatient Holter, VT on device interrogation and in hospital cardiac arrest

^**^The composite endpoint of clinically relevant ventricular arrhythmia included VT on telemetry requiring initiation of antiarrhythmic therapy, sustained VT (> 30 s) on telemetry, non-sustained VT > 10 s on device interrogation and in-hospital cardiac arrest

## Discussion

In this study, we analysed the prevalence, phenotypic characteristics, echocardiographic findings and clinical outcomes of MVP patients with and without MAD undergoing mitral valve surgery. Our main findings are that MAD is a common anatomical abnormality in patients undergoing surgery for MVP, with an incidence of 28.8%. We found patients with MAD were significantly younger, more likely to be female, with a lower incidence of hypertension, hyperlipidaemia and coronary artery disease. Anatomically, MAD patients were more likely to have myxomatous leaflets, bileaflet prolapse, longer anterior and posterior mitral valve leaflets, larger mitral annular end systolic and end diastolic dimensions. We demonstrated that MAD positive patients have excellent postoperative outcomes, with a high proportion of patients undergoing successful mitral valve repair, suggesting MAD does not compromise mitral valve surgical reparability. We found during a mean follow-up period of 3.91 years following surgery, that patients with MAD showed a signal towards higher rates of ventricular arrhythmia despite complete surgical correction of the disjunction, with no significant difference in postoperative mortality, postoperative mitral regurgitation or 30-day hospital readmission. This is hypothesis generating that arrhythmia risk may continue despite correction of disjunction, however larger studies with more robust arrhythmia monitoring and longer follow up are needed to conclusively answer this question.

The prevalence of MAD in patients with MVP has been reported with considerable variation in the literature depending on the definition and imaging modality used. A systematic review by Bennet et al. found the prevalence of MAD among patients with mitral valve prolapse independent of MR severity to be 32.6% [15]. More recently a large cohort of 595 patients found 31% of MVP patients had MAD, with 166 (28%) patients having severe MR. Of the severe MR subset 30% were found to have MAD, and no independent link between MAD and MR severity was observed [21]. To our knowledge the only recent literature looking at MAD in MVP patients undergoing MV surgery was described in a letter to the editor detailing a 16.9% prevalence of MAD among 838 patients undergoing MV surgery [18]. We found 28.8% of patients had concurrent MAD in the setting of MVP undergoing mitral valve surgery, which is consistent with previous studies and re-enforces that MAD is associated with MVP independent of MR severity.

In terms of the MAD phenotype in MVP patients undergoing surgery, we found MAD was significantly associated with younger age, lower rates of hypertension, hyperlipidaemia and coronary artery disease, myxomatous bileaflet prolapse and longer anterior and posterior leaflets similar to other recently published cohorts [21]. We also found a significant association between MAD and female sex which has been inconsistently described in previous trials [14, 19, 21]. It was previously reported that MVP is predominantly associated with female gender [25], thought to be due to referral bias, that women were more likely to present with symptoms compared to men, subsequently inflating the prevalence. Later studies ruled out this referral bias by studying community populations, that found women do in fact have a higher prevalence of MVP [26]. However, the reason for this remains unknown. Our cohort was 70.3% male, which is consistent with prior studies demonstrating that males with MVP are more likely to progress to severe MR requiring surgery [19]. Despite the high percentage of males in our study, women were significantly more likely to have MAD. This is consistent with the female predominance of MAD shown by Putnam et al. who analysed 90 patients with mitral valve prolapse and severe MR via cardiac computed tomography. They identified 18 patients (20%) with MAD and found that female gender was the parameter most strongly associated with disjunction [19].

We found excellent post-surgical mortality, post-surgical MR and hospital re-admission outcomes in both MAD positive and MAD negative patients in our cohort comparable to other post- surgical MVP trials and registry data [27–29] Our rates of MVP replacement were 19.8%, which is higher than other trials [21], possibly due to increased patient age and co-morbidities in our cohort, as well as surgical experience and preference at our centre. However, it is reassuring that 90.6% of MAD positive patients underwent successful repair, which suggests the presence of MAD does not compromise MV surgical reparability. Importantly we also found all patients in our cohort had complete correction of MAD post-surgery, likely because of the suturing of ring and prosthesis, joining the annulus to the LV myocardium, and collapsing the MAD gap [21]. This has potential important future implications as indication expansion and advances in transcatheter edge to edge mitral valve repair occurs, as transcatheter repair does not correct disjunction. However, whether preference should be given to surgical repair in patients with MAD and severe MR appears logical but has not yet been determined.

Previous studies have suggested that MAD is associated with an increased risk of ventricular arrhythmias [8, 9, 11]. It has been postulated that areas of MAD generate mechanical stress of the papillary muscles and ventricular endocardium, which over time can cause myocardial fibrosis in some patients [13]. A recent large cohort study by Essayah et al. demonstrated MAD presence with MVP is associated with progressive excess incidence of clinical arrhythmic events after diagnosis without an increase in mortality within the first 10 years, and they highlighted the importance of arrhythmia monitoring in the follow up of these patients [21]. In 2022 the European Heart Rhythm association published an expert consensus statement outlining a directed and structured risk stratification process aimed at identifying arrhythmic MVP and assessing the risk of sudden cardiac death [20], however the importance of ongoing arrhythmia monitoring after mitral valve surgery is not discussed.

We found a possible ongoing increased incidence of ventricular arrhythmic events in patients with MAD and MVP, despite complete correction of disjunction with surgery. This is consistent with the small subset of patients who underwent surgery in the paper described by Essayah et al [21]. This can possibly be explained by the fact that surgery corrects the disjunction, but myocardial fibrosis may have already occurred. These findings highlight the importance to both detect and report the presence of MAD in patients being referred for mitral valve surgery, to ensure the correct surgical technique to correct the disjunction, and to facilitate appropriate post-surgical arrhythmia monitoring similar to high risk MVP patients that have not undergone surgery.

This study has several limitations. Firstly, it is a single centre, retrospective cohort study with a small sample size. Although we retrospectively identified our cohort, patients were diagnosed consecutively and the ANZSCTS database was gathered prospectively which provided all demographic data and 30-day outcomes, however 4-year outcomes relied on retrospective review of medical records, specialist letters, Holter monitor and device interrogation reports. Echocardiographic images were reviewed and all measurements reported were performed by the investigator blinded to outcomes. MAD was reliably recorded in all patients due to routine acquision of a parasternal long axis image, however the pickelhaube sign was often uninterpretable due to cut off of the peak systolic spike. Secondly, due to the retrospective nature of the study there was a lack of robust, systematic arrhythmia monitoring, with outpatient arrhythmia monitoring only undertaken if there was a clinical indication, which led to a small number of patients (42/111, 38%) having a Holter monitor performed during follow up. Finally, myocardial fibrosis on cardiac MRI was not included in baseline demographics as it was not part of routine MVP evaluation at the time of the study, however other high-risk features such as inferior T-wave inversion on baseline ECG, and pickelhaube sign were recorded. There is potential for future studies to evaluate post-surgical arrhythmia risk in the subset of MVP patients with MAD and myocardial fibrosis on cardiac MRI.

## Conclusion

In conclusion, MAD is a common finding in patients with MVP undergoing surgery, and is associated with younger age, female gender, less cardiovascular risk factors, lower rates of coronary artery disease, and longer, myxomatous leaflets with bileaflet prolapse. Concurrent MAD does not compromise MV surgical reparability, and both repair and replacement are both effective at correcting disjunction. MAD presence in MVP patients undergoing surgery may potentially infer an ongoing arrhythmia risk despite correction of disjunction, with no difference in mortality, post-operative mitral regurgitation or hospital re-admission. Post-surgical arrhythmia monitoring similar to high risk MVP patients that have not undergone surgery may be appropriate however larger studies with more robust arrhythmia monitoring and longer follow up are needed to conclusively answer this question.

## Data Availability

The Data underlying this article will be shared on reasonable request to the corresponding author.
